# From PALSA PLUS to PALM PLUS: adapting and developing a South African guideline and training intervention to better integrate HIV/AIDS care with primary care in rural health centers in Malawi

**DOI:** 10.1186/1748-5908-6-82

**Published:** 2011-07-26

**Authors:** Michael J Schull, Ruth Cornick, Sandy Thompson, Gill Faris, Lara Fairall, Barry Burciul, Sumeet Sodhi, Beverley Draper, Martias Joshua, Martha Mondiwa, Hastings Banda, Damson Kathyola, Eric Bateman, Merrick Zwarenstein

**Affiliations:** 1 Dignitas International, 2 Adelaide Street West, Suite 200, Toronto, M5H 1L6, Canada; 2 Department of Medicine, University of Toronto, 200 Elizabeth Street, Toronto, M5G 2C4, Canada; 3 Department of Health Policy, Management and Evaluation, University of Toronto, 155 College Street, Suite 425, Toronto, M5T 3M6, Canada; 4 Clinical Epidemiology Unit, Sunnybrook Health Sciences Center, 2075 Bayview Ave, Toronto, M4N 3M5 Canada; 5 Knowledge Translation Unit, University of Cape Town Lung Institute, University of Cape Town, PO Box 34560, Groote Schuur 7937, South Africa; 6 Ministry of Health Malawi, POB 3, Lilongwe, Malawi; 7 Zomba Central Hospital, Kamuzu Highway, Zomba, Malawi; 8 Nurses and Midwives Council of Malawi, POB 30361, Lilongwe, Malawi; 9 Research for Equity and Community Health (REACH) Trust, POB 1597, Lilongwe, Malawi

## Abstract

**Background:**

Only about one-third of eligible HIV/AIDS patients receive anti-retroviral treatment (ART). Decentralizing treatment is crucial to wider and more equitable access, but key obstacles are a shortage of trained healthcare workers (HCW) and challenges integrating HIV/AIDS care with other primary care. This report describes the development of a guideline and training program (PALM PLUS) designed to integrate HIV/AIDS care with other primary care in Malawi. PALM PLUS was adapted from PALSA PLUS, developed in South Africa, and targets middle-cadre HCWs (clinical officers, nurses, and medical assistants). We adapted it to align with Malawi's national treatment protocols, more varied healthcare workforce, and weaker health system infrastructure.

**Methods/Design:**

The international research team included the developers of the PALSA PLUS program, key Malawi-based team members and personnel from national and district level Ministry of Health (MoH), professional associations, and an international non-governmental organization. The PALSA PLUS guideline was extensively revised based on Malawi national disease-specific guidelines. Advice and input was sought from local clinical experts, including middle-cadre personnel, as well as Malawi MoH personnel and representatives of Malawian professional associations.

**Results:**

An integrated guideline adapted to Malawian protocols for adults with respiratory conditions, HIV/AIDS, tuberculosis, and other primary care conditions was developed. The training program was adapted to Malawi's health system and district-level supervision structure. PALM PLUS is currently being piloted in a cluster-randomized trial in health centers in Malawi (ISRCTN47805230).

**Discussion:**

The PALM PLUS guideline and training intervention targets primary care middle-cadre HCWs with the objective of improving HCW satisfaction and retention, and the quality of patient care. Successful adaptations are feasible, even across health systems as different as those of South Africa and Malawi.

## Introduction

There has been substantial progress in improving access to antiretroviral treatment (ART) for people with HIV/AIDS, which are now estimated to number some 33 million [1]. ART initiations have increased yearly, yet worldwide, there are still more than 10 million ART-eligible HIV patients who are not receiving it [1]. In Malawi, a low income country in sub-Saharan Africa, almost one million out of a population of 14 million people are living with HIV[1], and it is a major factor behind the country's low life expectancy of just 43 years [2,3]. Recent data suggest that about 211,000 adults and children were alive and on ART [4]. As in several other resource-poor countries in sub-Saharan Africa, the government of Malawi has committed to further scale-up HIV/AIDS treatment [1,5]. One important strategy to successfully scale-up access to ART is to decentralize HIV/AIDS services to rural primary care centers [1,6].

However, scaling up access to ART and other health services while maintaining quality of care is a challenge given major shortages of trained healthcare workers (HCW): in Malawi, the HCW vacancy rate is 50% [7-9]. Innovative interventions and strategies are required to improve the use and training of existing human resources [8,10,11], and to address the fact that HIV/AIDS patients often have important co-morbidities like tuberculosis (TB) and malaria, highlighting the need for training of HCWs in the integrated management of HIV/AIDS, TB and other priority primary care diseases. Attention to the quality of clinical care provided while increasing access to HIV/AIDS services is also important, especially since the scale-up of HIV/AIDS services may have negative consequences on existing primary care services in those same centers [7,12].

Training strategies proven to work elsewhere may be useful in new settings, however they must be adapted to be consistent with local practice and policy, and be developed in collaboration with local experts and stakeholders [6]. One such strategy is the Practical Approach to Lung Health and HIV/AIDS in South Africa, or PALSA PLUS, adapted from the World Health Organization's Practical Approach to Lung Health [13]. In rigorous studies, the implementation of PALSA PLUS with nurses in health centers in South Africa demonstrated improved patient outcomes related to TB, asthma treatment, and HIV [14-16]. Nurses trained with PALSA PLUS reported better emotional and operational support from their outreach trainers, and increased confidence in integrating HIV/AIDS care with primary care [17,18], potentially important factors for improving staffing and strengthening the healthcare system.

PALSA PLUS combines primary care guidelines with educational outreach delivered by trained nurse-managers. This paper describes its adaptation for Malawi's primary care setting, where HIV/AIDS services are being decentralized. PALM PLUS was designed to integrate existing Malawian disease-specific guidelines into a single document for the primary care setting. PALM PLUS aims to support mid-level HCWs, *i.e.*, nurses and non-physician clinicians (clinical officers and medical assistants), to improve access to and quality of HIV/AIDS and primary care services. The primary objectives of PALM PLUS, now being implemented in Malawi, are to improve mid-level HCW job satisfaction and retention in rural health centres; secondary objectives are to improve quality of patient care.

## Methods

The process of adapting the PALSA PLUS guideline and training to Malawi began with the creation of an international and inter-professional team including representatives from the Knowledge Translation Unit of the University of Cape Town Lung Institute, the developers of the original PALSA PLUS guideline and training program [19]; Malawi-based members of Dignitas International (DI), the non-governmental organization that was intending to lead the implementation and evaluation of the Malawi version of the guideline; the Research for Equity and Community Health Trust (REACH Trust) who were partners in the evaluation; and the Malawi Ministry of Health (MoH) and the Malawi Nurses and Midwives Council.

### Adaptation of the clinical guideline

A two-phase review of PALSA PLUS content was carried out over a one-year period. In the first phase, priority conditions for inclusion in the Malawian adaptation were determined based on discussions with key national and district level MoH personnel, representatives of Malawian professional associations, clinicians working in primary and secondary care in Malawi, and representatives of DI.

The second phase involved a detailed review of the most current versions of Malawian national treatment policies. National disease-based (*e.g.*, ART, malaria, TB) and Standard Treatment Guidelines were obtained. The national guidelines are produced by technical expert committees appointed by the MoH and are revised regularly; recommendations are based on reviews of current evidence, international guidelines, and an assessment of local feasibility. We also consulted with representatives of the responsible Ministry Departments regarding any recent, but not yet published, changes to existing national policies and guidelines (Table 1).

**Table 1:** Malawi National Guidelines Consulted in the Development of PALM PLUS

Guideline Name	Edition/Date
Guidelines for the Use of Antiretroviral Therapy in Malawi	3rd Edition, April 2008

Malawi Standard Treatment Guidelines	4th Edition, 2009

National Tuberculosis Control Program Manual	6th Edition, 2007

Guideline for the Management of Malaria	August, 2007

Prevention of Mother to Child Transmission of HIV and Paediatric HIV Care Guidelines	2nd edition, July 2008

Management of HIV Related Diseases	2nd edition, April 2008

Guidelines for the Management of Sexual Assault and Rape in Malawi	November 2005

HIV/AIDS Counseling and Testing Guidelines For Malawi	2nd Edition, 2004

Management of Sexually Transmitted Infections Using Syndromic Management Approach	3rd Edition VI, March 2007

Malawi Essential Drug List	2009

The review process revealed inconsistencies across various guidelines. One example is the difference in the definition of fever: in the Malaria guideline the threshold for fever is not specified, while in the Standard Treatment Guidelines it is variably defined as '>38°Celsius', '>40.5°Celsius in adults', or '>38°Celsius, continuously or intermittently, for more than 24 hours in any 72 hour period' for patients with HIV. Another example relates to treatment recommendations when malaria is suspected, but a blood film is negative: the Malaria guideline recommends assessing for other causes of fever and does not recommend treating for malaria (there is no mention of considering HIV coinfection), whereas the HIV guideline recommends treating presumptively for malaria if the patient is known to be HIV positive. These inconsistencies were resolved in PALM PLUS based on available evidence, local expert opinion, and current best practices in Malawi.

Key guiding principles when considering the inclusion of specific content in the adapted guideline were ensuring the content's relevance in Malawi's primary care health centers, whether the diagnostic and therapeutic resources mentioned in the content were routinely available in such settings, and compliance with national guidelines, essential medicines list, and policy. Draft guideline sections were developed and reviewed by frontline clinicians (doctors, nurses, clinical officers, medical assistants), the relevant national MoH departments, and key leaders in the District Health Office, National MoH, and mission-run health centres given the importance of their input and the key roles they could play in the implementation of PALM PLUS. In an iterative process, detailed feedback was solicited, clarifications sought where required, and revisions submitted for further feedback. The reviewers were expected to check that the algorithms and content were correct and appropriate and reflected available drugs/resources in Malawian health centers. Some of the feedback was solicited and received via email, reducing the need for travel and ensuring participants could review material when convenient for them. This process aimed to ensure local relevance, to promote local ownership and to minimize barriers to implementation (*e.g.*, inadequate access to diagnostic resources listed in the guideline). Like PALSA PLUS in South Africa, PALM PLUS covers only the treatment of adults, but based on recommendations from MoH experts, we included references to the Malawian guideline for the management of children with HIV/AIDS, as well as a related key message (Table 2) and desk blotter illustration.

**Table 2:** Key Messages in PALM PLUS

Key Message	PALM PLUS Section(s) where key message appeared
Inhaled corticosteroids control asthma →13*	Asthma care

Smoking? Urge your client to stop.	Cough, COPD care, Using inhalers and spacers

Cough ≥3 weeks? Exclude TB →34*	Fever, Unwell, Lymphadenopathy, Cough, Treating HIV, Suspecting TB, Treating TB, Pregnancy

Prevent MDR/XDR TB. Urge adherence to TB treatment.	Suspecting TB, Treating TB

Status unknown? Test for HIV →28*	Unwell, Lymphadenopathy, Psychiatric, Headache, Cough, Ear, Throat/Mouth, Abdominal Pain, Diarrhoea, Rash, Pregnancy

Prevent AIDS with routine HIV care →29*	Psychiatric, Diarrhoea, Diagnosing HIV, Routine HIV Care, Treating HIV, ART Follow-up, PMTCT, Treating TB

HIV? Manage client and family.	Diagnosing HIV, Routine HIV Care, Treating HIV, PMTCT

Pregnant with HIV? Give PMTCT, routine ante natal and HIV care.	Routine HIV Care, ART Follow-up, PMTCT, Pregnancy

STI? • Educate about STI • Urge adherence • Treat partner/s • Give condoms • 'Stick to 1 partner' • Test for HIV	Lymphadenopathy, Female Genital Symptoms, Genital Ulcer Syndrome, Other Genital Problem, Pregnancy

Fever ≥3 weeks? Exclude malaria →1, then TB →34*	Fever, Unwell

*Indicates page number in PALM PLUS guideline to turn to for more information related to key message

### Adapting the training program

PALSA PLUS utilises a Train-the-Trainer-to-Train (TtTtT) approach where nurse middle managers are equipped with group facilitative skills, and familiarized with the content of the guideline, to enable them to deliver group educational outreach [14] training to all primary care staff at a facility during short (1 to 1.5 hour) sessions over several months. The TtTtT workshop is an intensive week-long live-in training during which managers are trained as facility trainers (Table 3). They are provided with multiple opportunities to experience receiving and delivering group facilitative training sessions using the guideline. A series of case scenarios is depicted by a waiting room scene, providing a structure for these otherwise less formal trainings, and ensuring that all critical guideline content is covered during the workshop. The training is grounded in adult education principles, and depends heavily on experiential learning and reflective practice in a group social context to facilitate development of training skills and familiarity with guideline content. Didactic content is minimal: the training includes a single powerpoint presentation used during the introductory session to provide a program overview. The training is supported by a desk blotter containing a two-year calendar to encourage clinicians to provide dates for follow-up appointments, and illustrated key messages and checklists (*e.g.*, routine HIV care) from the guideline.

**Table 3:** PALM PLUS Training the Trainer to Train (TtTtT) case scenario training plan

Day	Session	Description
Sun		Travel to venue

	1	Introduction to TtTtT PALM PLUS

Mon	1, 2	Straightforward symptom scenarios: • Cough and difficulty breathing -severe pneumonia • Runny/blocked nose - URTI • Blood in urine - Bilharzia • Confused patient - delirium • Fever - Malaria

	3,4	HIV testing • Headache - sinusitis • Approach to STI • Lymphadenopathy - Bubo • Vaginal discharge/sexually abused

Tues	1	Managing HIV - routine care & ART • Weight loss & sore mouth - focus on routine care • Asymptomatic - oral thrush - focus on starting ARVs • Diarrhoea - focus on ART follow-up

	2	TB - diagnosing, treatment & follow-up • Cough - HIV negative, sputum positive - focus on TB follow up • Fever - HIV unknown, 1 smear positive - educate about Malaria

	3	TB and HIV - diagnosing and treating both TB & HIV • Discharging ear - HIV positive, 1 smear positive - TB care • Cough - HIV positive, smear negative - Health worker with TB & HIV

	4	ART and/or TB drug side effects • Burning feet - peripheral neuropathy TB med related • Abdominal pain - drug related - drug related hepatitis - NVP or TB treatment • Vomiting - lactic acidosis - ARV side effect - ARV care • Skin rash - ARV side effect

Weds	1	Pregnancy: Unwell and tired - HIV pregnancy/PMTCT

	2	Chronic respiratory disease: Cough, difficult breathing with wheeze - acute asthma

	--	Free time

Thurs	--	Full day training experience - consolidation of content knowledge and training skills

Fri	1	Making PALM PLUS your own - a creative exercise

	2	Evaluation & Closure

Adaptation of the training program to Malawi focused on customization of the training materials, selection of outreach trainers, and adjustments to the TtTtT program. Customization of the training materials required commissioning new artwork for the waiting room scene and desk blotter to ensure local references were relevant to Malawi. The artist was provided with photographs of Malawians accessing care at health centres, and created a waiting room scene that would resonate with Malawian frontline healthcare workers. Selected illustrations from the desk blotter were replaced to better reflect Malawian patients, local drug packaging and commonly available diagnostic tests. The desk blotter's key messages were revised to reflect Malawian priorities, and case scenarios were reworked to be consistent with the adapted guideline (Table 3). Because of reduced access to investigations and essential medicines in Malawi as compared with South Africa, each scenario was adapted to include consideration of system constraints. In some instances, this allowed provision for these constraints in the guideline to be highlighted, such as how to manage a febrile patient in the absence of malaria test kits. In others, such as the lack of basic equipment like thermometers, the intention was rather to generate a clinic level discussion as to how they might start to address such deficiencies, for example lobbying the relevant supervisors to provide equipment.

Finally, two training manuals were adapted for the Malawian context: the master trainers' manual, providing instruction on how to train; and an implementation toolkit aimed at middle managers and trainers to clarify training implementation, onsite training, monitoring and evaluation, and responsibilities for implementation.

The selection of trainers was also adapted. Whereas in South Africa the trainers were nurse-managers trained to train other nurses, in Malawi, primary care health centers are staffed by a combination of nurses, clinical officers (three years of training plus a one-year internship) and/or medical assistants (two years of training and a one year internship). Given the severe health human resource shortage in Malawi [7,8], all three cadres work interchangeably with similar clinical duties in most primary care centers, and peer-trainers were chosen from all three cadres. Unlike in South Africa, the local District Health Office could not reliably provide transport for trainers, so they were chosen from the staff working at sites implementing PALM PLUS training, minimizing the need for travel to other sites. A total of 14 HCWs took part in the training program.

## Results

In the first phase, a draft contents page was created based on common key symptoms in primary care. The starting point was the contents in PALSA PLUS: asthma and chronic obstructive lung disease (COPD), HIV/AIDS, TB and sexually transmitted infections (STIs). Malaria was added given its epidemiological importance in primary care in Malawi. The PALSA PLUS guideline was designed for clinicians to use based on one of three 'entry-points' (table 4): a respiratory or STI chief complaint alone (*e.g.*, cough for less than two weeks); a known disease plus a new complaint (*e.g.*, HIV with headache); or specific diagnosis, treatment, or follow-up guidelines in the management of asthma, COPD, TB, HIV and STIs. The PALM PLUS guideline was modified to include only two 'entry-points': an expanded list of chief complaints in alphabetical order (with or without a co-morbidity) and specific diagnosis, treatment, and follow-up guidelines for the management of asthma, COPD, TB, HIV and STIs, as well as routine antenatal care (Table 5). The symptom algorithms integrated multiple possible common causes for each symptom; for example, the 'fever' page prompted the clinician to consider diagnoses of malaria, TB, or HIV as appropriate. Highlighted and distinct integrative key messages (short, summary information vectors) adapted to Malawi were included; these are simple and provide a reminder to integrate separate clinical algorithms (*e.g.*, on 'generalized red rash' algorithm, key message states 'Status unknown? Test for HIV page 28'). The final guideline comprised 44 pages.

**Table 4:** PALSA PLUS organization and content

Domain	Symptom-based algorithms	
Respiratory system	Cough and/or difficulty breathing <2 weeks with...	Wheezing/tight chest Sputum production and/or fever and/or pain on breathing/coughing Runny/Blocked nose Pain and tenderness over sinuses Sore Throat and/or mouth Ear problem
	
	Cough and/or difficulty breathing > = 2 weeks with...	Asthma (TB excluded) COPD (TB excluded) Chronic cough (TB/asthma/COPD excluded) Difficulty breathing(TB/asthma/COPD excluded)
Genito-urinary	Urethritis	
	Scrotal swelling or pain	
	Balanitis	
	Vaginal discharge	
	Lower abdominal pain without vaginal discharge	
	Genital ulcer syndrome	
	Other STIs	

HIV with...	Cough and/or difficulty breathing	
	Weight loss	
	Skin rashes	
	Headache	
	Eye problems	
	Burning feet	
	Vomiting	
	Diarrhoea	
	Abdominal pain (without diarrhoea)	
	Psychiatric symptoms	

**Domain**	**General clinical management algorithms**	

Tuberculosis	Suspecting TB	
	Diagnosis	
	Treatment	
	Follow-up	

Sexually Transmitted Infections	Approach to a client following sexual abuse	
	Approach to a client with an STI	
	Approach to the partner of a client with STI	
	Cervical screening	
	Positive syphilis result	

HIV	Diagnosis	
	Staging	
	Routine care	
	ARV initiation	
	ARV follow-up and side effects	
	PMTCT	

**Table 5:** PALM PLUS organization and content

Symptom based algorithms (alphabetical)							
A	Abdominal pain	F	Fatigue	M	Malaise	T	Throat symptoms

	Abdominal swelling		Female genital symptoms		Male Genital symptoms		Tiredness

B	Burning feet		Fever		Mouth symptoms	U	Unwell client

C	Confusion		Foot symptoms	N	Nose symptoms		Urinary symptoms

	Cough	G	Male genital symptoms	P	Psychiatric symptoms	V	Vaginal discharge

D	Diarrhea		Other genital symptoms	R	Raped client		Vomiting

	Difficulty breathing		Genital ulcer	S	Sexual abuse	W	Weight loss

	Disturbed client	H	Headache		Skin symptoms		

E	Ear symptoms	L	Lymphadenopathy				

**Domain**		**General clinical management algorithm**					

HIV		Diagnosis					
		Routine care					
		ARV initiation					
		ARV follow-up and side-effects					
		PMTCT					

Tuberculosis		Suspecting TB					
		Diagnosis					
		Treatment					

Chronic Respiratory Disease		Distinguishing asthma and COPD					
		Routine asthma care					
		Routine COPD care					
		Using inhalers and spacers					

Pregnancy		Routine antenatal care					

Fourteen trainers were identified through discussions with the Zomba District Health Office and the Catholic Health Commission based on the following criteria: nurses/clinical officers/medical assistants with sound relationships with their colleagues; knowledge of HIV/AIDS/TB; experience in rural primary care health centers; currently working at the health center they would train at or able to easily travel there; and willingness to attend training follow-up meetings. Prior experience in delivering training was not required.

The PALM PLUS TtTtT course was modified to reflect the flow of the PALM PLUS guideline and to provide sufficient understanding of the step-by-step approach in the guideline. Multiple opportunities to practice the methodology of interactive training were provided to increase confidence during onsite training. The structure of the training programme provided a safe learning environment that allowed respect of the social and cultural norms of Malawian learners. Professional and gender-based hierarchies (*i.e.*, clinical officers are more senior to other cadres, and are mostly men) in Malawi's social structure were respected; for example, clinical officers were invited to speak or give feedback first in small groups.

### Ethical considerations

The guideline and training adaptation was carried out as part of an intervention cluster-randomized trial [20]. The study has been approved by the National Health Sciences Research Committee, Malawi's national research ethics board.

## Discussion

This report describes the development of PALM PLUS, a single set of Malawi-adapted, integrated symptom- and sign-based primary care guidelines for adults, and an innovative training program for HCWs. PALM PLUS is not designed to replace national disease-specific guidelines, but rather to assist nurses and clinicians at health centers to integrate and apply existing guidelines and protocols more effectively. While we were successful in developing an integrated guideline tool to assist front-line HCWs, we are not yet able to say whether PALM PLUS is improving health outcomes. Clinical outcomes are being evaluated in a cluster-randomized-controlled trial (cRCT) of PALM PLUS guidelines in 30 rural health centers in a single district in Malawi, with approximately 200 HCWs in each arm of the trial.

Health system capacity and a shortage of trained workers have emerged as serious obstacles to achieving universal ART coverage [7-11]. The goal of ensuring equitable access to quality healthcare is further frustrated by the difficulty of retaining staff in rural areas of low- and middle-income countries (LMICs) [8]. Overcoming these obstacles requires innovative strategies to optimize the use of existing staff, and interventions to train and retain staff [8]. However, few studies have compared different interventions [21], and there is little evidence that is of direct use to the policymakers crafting health systems interventions [8].

Current models of HCW resource needs often look at HIV/AIDS care in isolation [7,22], without considering the need to provide other care, such as for co-morbidities or non-HIV primary care. The risks of a vertical approach to health services are known [23], however the push for rapid scale-up and decentralization of HIV/AIDS services, the lack of integration with primary care, and the potential for additional disease-specific vertical programs [24] makes integration at the primary care level even more pressing. Some integration of health training has occurred [25-27], yet integration of clinical services is often ineffective, incomplete or non-existent, especially with respect to HIV/AIDS care and women's reproductive health [28-31]. A recent study from Malawi found that 81% of HIV positive mothers enrolled in a Prevention of Mother to Child Transmission (PMTCT) program were lost to follow-up by the six-month post-natal visit [31], suggesting an urgent need for better integration of pre and post-natal maternal health services. A review of 25 countries with the highest HIV prevalence rates found that nearly all reported low national programme performance in controlling HIV-related TB, and called for closer integration of TB and HIV programmes [32]. Similar arguments have been made for malaria and HIV [33]. Yet few interventions designed to achieve integration of clinical care for front-line HCWs have been carefully evaluated.

Understanding the impact of integrated interventions on HCWs is therefore required. Several studies from Malawi suggest the importance of training and supervision to health provider retention [34-36]. Focus group discussions with HCWs in rural health centers identified opportunities for training and career progression, and weaknesses in clinical and district-level supervision as key factors affecting job retention [34]. Poor supervisory support and inadequate training resources for their clinical environment correlated with the likelihood of leaving the job and/or plans to leave the job within the next 12 months [35]. A comprehensive literature [8] review of health staff recruitment and retention in LMICs found that training and continuing education opportunities and management support affected retention in remote rural areas, especially in Africa, while better salaries were a cause of staff mobility for only one-quarter of respondents in those countries. The same review suggests that policy options to improve recruitment and retention in remote rural areas included improving training for rural practice and better clinical tools to improve working conditions [8].

PALM PLUS and PALSA PLUS seek to address these issues through the implementation and evaluation of a targeted intervention to optimize the clinical effectiveness of frontline healthcare workers in rural health centers in addressing HIV/AIDS, TB and priority primary care conditions. Recommendations in multiple national guidelines may be impossible to implement in small health centers due to lack of access to recommended tests or treatments, or even the guidelines themselves, at the primary health centre level. Traditional in-service training is often also disease-specific [24]. Disease-specific guidelines and training may be appropriate at specialized clinics in larger centers, but they provide limited support to front-line nurses and clinicians in primary care health centers. Clinical integration has begun to occur in Malawi, such as for TB and HIV/AIDS, but more comprehensive adult integrated guidelines and tools to assist the nurse or clinician in the consulting room have yet to be developed. Furthermore, the PALM PLUS guideline provides for greater empowerment of HCWs at the local level.

Our methodology was adapted from the original method to develop PALSA guidelines [37], and was similar to the process for adapting the Integrated Management of Childhood Illness (IMCI) guidelines, described by WHO some years ago [38]. Other approaches have been described, such as proposed by the ADAPTE group [39], which include explicit and systematic search for and grading of available evidence. Consistent application of such an approach, while laudable, would have been extremely resource-intensive and impracticable in our context given that we were developing an integrated guideline covering a large number of conditions. Secondly, a large component of the ADAPTE methodology includes deciding which guidelines to draw upon. When adapting a guideline for a public health setting, this process is replaced by what we did -- source all relevant national and local guidelines, review for consistency, identify 'red-flags' (areas where we may be concerned about the evidence-basis for the recommendations and recommendations that do not account for local resource constraints) -- and work with Ministry partners to find solutions that are consistent with evidence, but can also be feasibly implemented. These differences speak to the underlying intention of our process versus conventional guideline development processes, where the motivation is to review how a condition is diagnosed and treated. Our motivation is rather to bring existing national guidelines together into a cohesive simplified easy-to-use tool that renders them implementable by variably skilled health workers working in constrained services. Our process, while less rigorous in terms of rating guidelines and recommendations in terms of the evidence they draw on, places more emphasis on ensuring the adaptation is compliant with country policies.

Our success at developing the PALM PLUS guideline and training program for Malawi's specific context suggests that it is possible to adapt it for use in other resource-poor settings. Qualitative evaluation of HCW perceptions of the PALM PLUS guideline and training, being carried out as part of cRCT implementation trial, will provide important data and lessons from this experience and may provide guidance for future adaptations. To date, these lessons include having at least one partner organization which takes a primary responsibility for leading the process (a non-governmental organization in the case of PALM PLUS though this could also be a governmental body), support from the original team, having staff dedicated to guideline development, involvement of ministry and key opinion leaders from early on in the process, and working to resolve any conflicts with other program priorities. The on-site intermittent training utilized in PALM PLUS reduced the cost and complexity of the training program by limiting the need for transportation and allowed for training to be scheduled when convenient for both trainers and trainees. Staff did not need not leave their patients, colleagues, or families behind for days or weeks in order to undergo training, unlike in off-site training programs. However, some HCWs may perceive this as a disadvantage, since off-site training can be seen as a break from the daily grind of care delivery in remote health centers, and the per diems that usually accompany off-site training may be of substantial value to staff. This question is being formally assessed in a qualitative evaluation being carried out as part of the PALM PLUS implementation. We did not include patients in the development of the guidelines, because to do so in Malawi presented tremendous challenges including language, limited healthcare fluency among representative patients, and cultural hierarchical barriers limiting opportunities for patients to challenge the views of HCWs. However, we are evaluating the effect of the PALM PLUS guidelines in healthcare-worker/patient clinical interactions through direct observation as part of our cluster randomized trial.

There were costs associated with the development of PALM PLUS, and there would be costs with their adaptation for other countries, but such costs may need to be seen as an integral part of a commitment to expand access to ART, such has been done by Malawi. A formal costing of the development of the PALM PLUS guidelines is part of our evaluation. While health system resources and structures in South Africa and Malawi are substantially different, the broadly similar disease burden combined with their geographic proximity may allow for easier adaptation. This may result in easier adaptation within the African continent than to other developing countries elsewhere.

## Competing interests

The authors declare that they have no competing interests.

## Authors' contributions

MS, MZ, EB, SS, BB, and ST conceived the project. MS, SS, and BB led grant development. RC, ST, and LF led the guideline adaptation. GF and ST were responsible for adapting the training curriculum. DK, HB, MM, and MJ helped design implementation, evaluation, and content. MS led the manuscript writing. All authors approved the final manuscript.
